# The Association between State Policy Environments and Self-Rated Health Disparities for Sexual Minorities in the United States

**DOI:** 10.3390/ijerph15061136

**Published:** 2018-06-01

**Authors:** Gilbert Gonzales, Jesse M. Ehrenfeld

**Affiliations:** 1 Department of Health Policy, Vanderbilt University School of Medicine, Nashville, TN 37203, USA; 2 Departments of Anesthesiology, Biomedical Informatics, Surgery & Health Policy, Vanderbilt University School of Medicine, Nashville, TN 37232, USA; jesse.ehrenfeld@vanderbilt.edu

**Keywords:** LGBT health, health policy, state policy, health disparities

## Abstract

A large body of research has documented disparities in health and access to care for lesbian, gay, and bisexual (LGB) people in the United States. Less research has examined how the level of legal protection afforded to LGB people (the state policy environment) affects health disparities for sexual minorities. This study used data on 14,687 sexual minority adults and 490,071 heterosexual adults from the 2014–2016 Behavioral Risk Factor Surveillance System to document differences in health. Unadjusted state-specific prevalence estimates and multivariable logistic regression models were used to compare poor/fair self-rated health by gender, sexual minority status, and state policy environments (comprehensive versus limited protections for LGB people). We found disparities in self-rated health between sexual minority adults and heterosexual adults in most states. On average, sexual minority men in states with limited protections and sexual minority women in states with either comprehensive or limited protections were more likely to report poor/fair self-rated health compared to their heterosexual counterparts. This study adds new findings on the association between state policy environments and self-rated health for sexual minorities and suggests differences in this relationship by gender. The associations and impacts of state-specific policies affecting LGB populations may vary by gender, as well as other intersectional identities.

## 1. Introduction

Numerous studies have documented wide disparities in health status for sexual minorities, or gay, lesbian, bisexual (LGB), and other nonheterosexual populations [1,2,3,4,5,6,7]. First, sexual minorities experience barriers to routine medical care. Numerous studies have noted higher levels of uninsurance and reports of financial barriers to care for LGB people compared with their heterosexual peers [8,9]. Many employers historically did not cover same-sex spouses of sexual minority workers—leaving some LGB individuals without health care benefits. Additionally, sexual minorities are more likely to report worse health status compared to their heterosexual counterparts due to, in part, “minority stress”, which is the additional stress sexual minorities experience associated with being a member of a marginalized community [10,11,12,13]. Discriminatory environments can stigmatize sexual minorities and reduce their self-esteem and confidence, which may lead to adverse health and risk-taking behaviors. Previous research has noted that sexual minorities living in states without legal protections (e.g., same-sex marriage) were more likely to report symptoms of depression, anxiety, and alcohol use disorder [14,15].

A growing body of research has also shown that the state policy environment for sexual minorities, defined as the legal protections afforded, is related to health. Rooted in social ecological theories of health [16], public policies can have a downstream effect on population health, health behaviors, and resources needed for better health. For instance, living in a state with legal same-sex marriage was associated with better self-rated health for individuals in same-sex relationships in the 2010–2013 Current Population Survey [17]. Meanwhile, living in a state with legal protections against hate crimes and employment-based discrimination for sexual minorities was associated with reduced prevalence of depression and anxiety disorder for sexual minorities participating in the 2004–2005 National Epidemiological Survey on Alcohol and Related Conditions [18]. More recently, a study using data from the 2011–2015 Behavioral Risk Factor Surveillance System found that living in a state with public attitudes and laws supporting legal protections for sexual minorities was associated with better self-rated health among lesbian and gay participants; however, this study did not stratify the analysis by gender or make comparisons to heterosexual individuals [19]. Other studies using samples from single states have found same-sex marriage laws to be associated with improvements in health insurance coverage [20,21] and reductions in mental health service utilization [22].

This study therefore builds on previous research and extends the analysis of self-rated health disparities by gender and sexual minority status. We hypothesize that living in a state with comprehensive legal protections for sexual minorities is associated with narrower disparities in self-rated health for lesbian, gay, and bisexual individuals. To test this hypothesis, we used recently collected data from a large, population-based, and multistate survey to compare self-rated health by state policy environment (comprehensive versus limited protections for LGB people). While previous studies have found that self-rated health for sexual minorities was generally better in states with legal protections, early studies often lacked a comparison group of heterosexual individuals to examine disparities in self-rated health. This study examines (1) how disparities in self-rated health vary across the United States and (2) whether disparities in self-rated health are modified by comprehensive legal protections for sexual minorities.

## 2. Materials and Methods

This study analyzed recently collected data from the 2014–2016 Behavioral Risk Factor Surveillance System (BRFSS). The BRFSS is a nationally representative and telephone-based survey of civilian, noninstitutionalized adults aged 18 years and older in the United States. Conducted since 1984 by the Centers for Disease Control and Prevention (CDC) in coordination with state health departments, the BRFSS now collects health information for approximately 450,000 randomly selected adults each year. The core questionnaire ascertains key demographic and socioeconomic characteristics, medical diagnoses, health behaviors, and access to medical care. States can participate in optional CDC-designed modules or add their own specific questions to their statewide BRFSS surveys. The median response rate across all states was 47.1% in 2016 (which ranged from 30.7% in Louisiana to 65.0% in Wyoming) [23].

The core demographic questionnaire in the BRFSS currently does not ask respondents about their sexual orientation, but some states have previously added sexual orientation questions to their individual BRFSS surveys [2,24,25]. Because responses to state-added questions are not submitted to the CDC, analyzing health and access to care for sexual minorities in previous years required approval from each individual state [4]. Beginning in 2014, however, the CDC allowed states to add and submit their responses to an optional and unified module that asked about sexual orientation and gender identity. The following 31 states added the sexual orientation and gender identity module to their BRFSS survey in 2014, 2015, and/or 2016: California, Colorado, Connecticut, Delaware, Georgia, Hawaii, Idaho, Illinois, Indiana, Iowa, Kansas, Kentucky, Louisiana, Maryland, Massachusetts, Minnesota, Mississippi, Missouri, Montana, Nevada, New York, Ohio, Pennsylvania, Rhode Island, Texas, Vermont, Virginia, Washington, West Virginia, Wisconsin, and Wyoming.

In the sexual orientation and gender identity module, respondents were asked which of the following categories best represents how they identify themselves: straight; lesbian or gay; or bisexual. While the BRFSS does include information on transgender status, we chose to focus this study on the association between state policy environments and self-rated health for sexual minorities. Our final sample included 14,687 sexual minority adults aged 18 years and older and 490,071 heterosexual adults aged 18 years and older. Our analysis excluded respondents indicating their sexual orientation as something else (*n* = 1945), respondents who did not know the answer (*n* = 5117), respondents who refused to answer (*n* = 8961), and respondents missing information on self-rated health (*n* = 1521). Previous research on nonresponse to a related sexual orientation question in state-level BRFSS surveys reported higher prevalence of nonresponse among racial and ethnic minorities [26], but nonresponse to a sexual orientation question was similar to nonresponse in other sensitive questions, such as race, ethnicity, and body weight [27].

We used descriptive statistics and chi-square tests to characterize the study sample. We then estimated the prevalence of poor/fair self-rated health (versus excellent, very good, or good health) by sexual minority status for all 31 states ascertaining sexual orientation. We restricted state-specific estimates to states with 50 or more sexual minority respondents to ensure a large enough sample size (28 states for men; 30 states for women). Then, we compared poor/fair self-rated health by sexual minority status and state policy environments. Individuals from all 31 states were classified into two categories based on the level of legal protections granted to sexual minorities in 2014 (the first year when the Human Rights Campaign collected data on the following five policies affecting sexual minorities) [28]. More specifically, we considered states to have comprehensive legal protections if they enacted all five of the following policies that are inclusive of sexual orientation: hate crime protections, legal same-sex marriage, employment nondiscrimination, nondiscrimination in housing, and nondiscrimination in public accommodations. Of the 31 states participating in the optional sexual orientation module, 16 states had comprehensive protections for sexual minorities by 2014 (CA, CO, CT, DE, HI, IL, IA, MD, MA, MN, NV, NY, RI, VT, WA, and WI) [28]. The remaining 15 states were classified as states with limited or no legal protections (GA, ID, IN, KS, KY, LA, MS, MO, MT, OH, PA, TX, VA, WV, and WY). While some policies have changed during the study period, especially with the recognition of federal same-sex marriage, all 15 of the study states with limited protections still lacked at least one of these protections routinely monitored by the Human Rights Campaign throughout the study period [29,30].

Unadjusted and adjusted logistic regression models were conducted to compare poor/fair health by sexual minority status, first for all adults and then stratified by state policy environments (comprehensive versus limited legal protection). All analyses were conducted separately by gender. Our fully adjusted models controlled for age, race/ethnicity, relationship status, the presence of a child in the household, educational attainment, employment status, household income, chronic disease diagnoses, health insurance status, having a usual source of care, state of residence, and survey year. Results from the logistic regression models are presented as odds ratios (ORs) with 95% confidence intervals (CIs). All analyses were conducted in Stata version 14 using survey weights and the *svy* command to reflect the complex survey design of the BRFSS. This study was deemed exempt by the Institutional Review Board because data were obtained from publicly available and secondary sources.

## 3. Results

Table 1 presents demographic and socioeconomic characteristics of the sample by gender and sexual minority status. Approximately 3.6–3.8% of respondents self-identify as lesbian, gay, or bisexual. Consistent with prior studies [31], sexual minority men were more likely to be younger (over 40% of sexual minority men were under 35 years of age compared to 29% of heterosexual men), racially and ethnically diverse, and college graduates. Sexual minority men were less likely to be married or living with a partner, less likely to have a child in the household, less likely to be employed, and less likely to have household incomes of $50,000 or more compared to their heterosexual peers. Although sexual minority men were slightly less likely to report a chronic disease diagnosis, there were no statistical differences in health insurance status and having a usual source of care.

Table 1 also presents the sociodemographic characteristics for sexual minority women and heterosexual women. Again, consistent with prior studies [31], sexual minority women were more likely to be younger (over half of sexual minority women were under 35 years of age compared to 25% of heterosexual women) and racially and ethnically diverse. Sexual minority women were also less likely to be married or living with a partner, but they were more likely to have a child in the household compared to heterosexual women. Educational attainment was relatively similar between sexual minority and heterosexual women, but sexual minority women were more likely to be unemployed and in households with incomes less than $50,000. Compared with heterosexual women, sexual minority women were less likely to report a chronic disease diagnosis, less likely to have health insurance, and less likely to have a usual source of care.

### 3.1. State-Specific Disparities in Self-Rated Health

**Figure 1:**
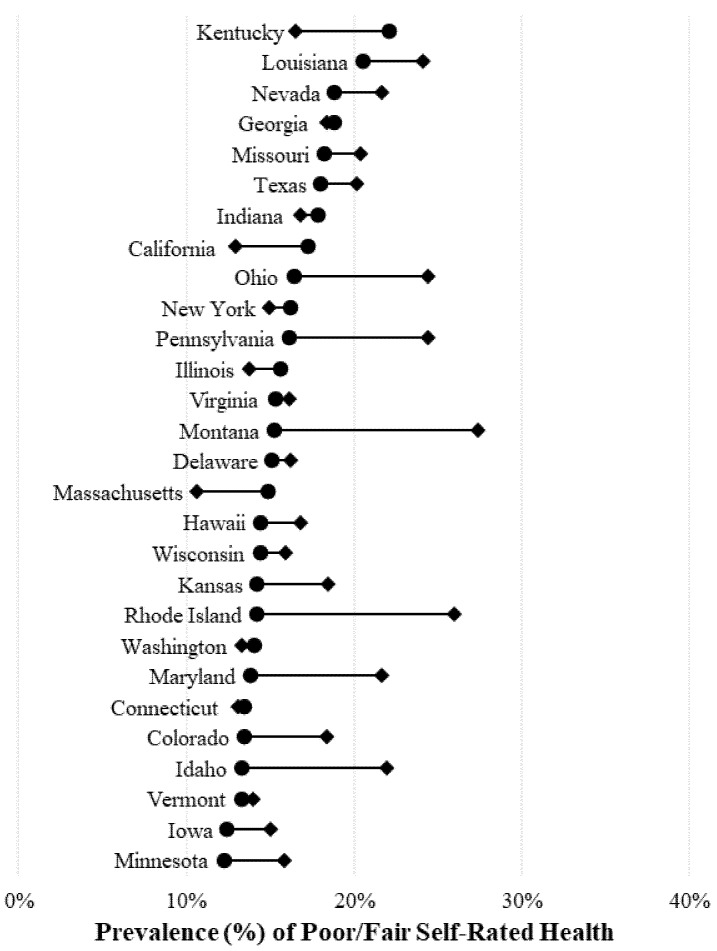
State-specific estimates of poor or fair health by sexual minority status for men. Weighted estimates represent the percentage of adults aged 18 years and older who report poor or fair health (instead of excellent, very good, or good health). ● = heterosexual adults; ♦ = sexual minority adults. Source: 2014–2016 Behavioral Risk Factor Surveillance Survey. Note that not all states in the figure ascertained sexual orientation every year, but comparisons between heterosexual and sexual minority adults are state–year specific.

Figure 1 presents the unadjusted state-specific estimates of poor/fair self-rated health by sexual minority status for men. Sexual minority men were more likely to report poor/fair health compared to heterosexual men in most states. The widest disparities in poor/fair health for sexual minority men were found in Montana and Rhode Island—where sexual minority men were, on average, 10 percentage points more likely to report poor/fair health compared with heterosexual men. Reporting of poor/fair health among sexual minority men was also high in Pennsylvania, Ohio, Louisiana, Idaho, Nevada, and Maryland; more than 20% of sexual minority men in these states reported poor/fair health. Sexual minority men were less likely to report poor/fair health in seven states: California, Illinois, Indiana, Kentucky, Massachusetts, New York, and Washington.

**Figure 2:**
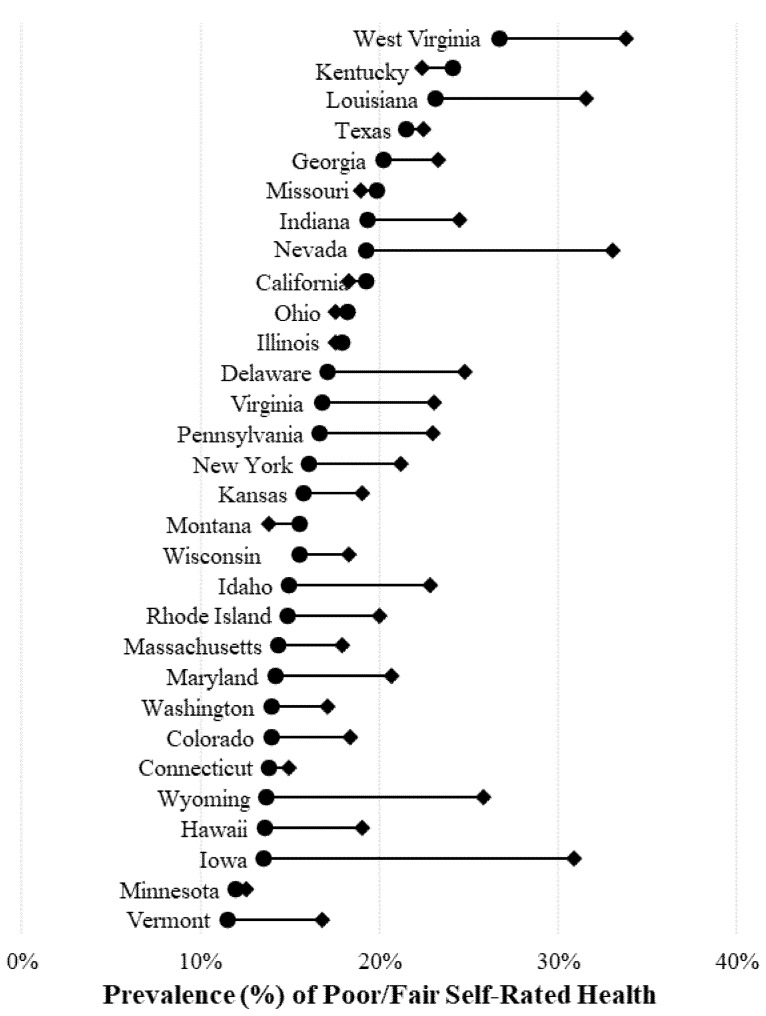
State-specific estimates of poor or fair health by sexual minority status for women. Weighted estimates represent the percentage of adults aged 18 years and older who report poor or fair health (instead of excellent, very good, or good health). ● = heterosexual adults; ♦ = sexual minority adults. Source: 2014–2016 Behavioral Risk Factor Surveillance Survey. Note that not all states in the figure ascertained sexual orientation every year, but comparisons between heterosexual and sexual minority adults are state–year specific.

Figure 2 presents the unadjusted state-specific estimates of poor/fair self-rated health by sexual minority status for women. Sexual minority women were also more likely to report poor/fair health compared with heterosexual women in most states. Disparities in poor/fair health by sexual minority status were widest in Iowa, Nevada, and Wyoming—all states where sexual minority women were more likely to report poor/fair health compared with heterosexual women by 10 percentage points or more. Sexual minority women in West Virginia, Nevada, and Louisiana also reported high levels of poor/fair health; more than 30% of sexual minority women living in these states reported poor/fair health. Sexual minority women in California, Kentucky Missouri, Montana, and Ohio were less likely to report poor/fair health compared with heterosexual women.

### 3.2. Association between State Policy Environments and Self-Rated Health Disparities

Table 2 presents the prevalence and odds ratios of poor/fair health among men by sexual minority status and state policy environments. There were no statistical differences in self-rated poor/fair health between sexual minority (17.4%) and heterosexual (16.4%) men in all study states—even after controlling for sociodemographic characteristics (odds ratio [OR] = 1.10; 95% confidence interval [CI] = 0.94–1.28). After stratifying by state policy environments, there were still no differences in self-rated poor/fair health for sexual minority (14.9%) and heterosexual (15.4%) men in the study states with comprehensive legal protections for sexual minorities (OR = 0.96; 95% CI = 0.79–1.16). However, sexual minority men in states with limited protections were more likely to report poor/fair health compared with heterosexual men (20.6% vs. 17.4%). This relationship persisted after controlling for sociodemographic variables (OR = 1.30; 95% CI = 1.03–1.64).

Table 3 presents prevalence estimates and odds ratios comparing poor/fair health among women by sexual minority status and state policy environment. On average, sexual minority women (20.7%) were more likely to report poor/fair health (OR = 1.21; 95% CI = 1.09–1.35) compared with heterosexual women (17.7%) after controlling for sociodemographic characteristics. Additionally, sexual minority women were more likely to report poor/fair health in states with comprehensive protections (OR = 1.26; 95% CI = 1.08–1.46) and in states with limited protections (OR = 1.19; 95% CI = 1.01–1.40). State policy environments did not modify the association between sexual minority status and self-rated health for women in this analysis.

## 4. Discussion

This study used a well-validated, population-based, and multistate sample of sexual minority and heterosexual adults in the United States to document disparities in self-rated health by state policy environment. We tested whether living in a state with comprehensive legal protections for sexual minorities (i.e., hate crime protections, legal same-sex marriage, employment nondiscrimination, nondiscrimination in housing, and nondiscrimination in public accommodations) was associated with narrower differences in self-rated health by sexual minority status. As hypothesized, we found narrower and nonsignificant differences in self-rated health for sexual minority men in states with comprehensive protections, but sexual minority men in states with limited protections were more likely to report poor/fair self-rated health compared with heterosexual men. Unexpectedly, we did not find a similar relationship for sexual minority women, who were more likely to report poor/fair self-rated health regardless of the state policy environment.

This study is consistent with previous research that has documented the association between better self-rated health among same-sex couples [17], sexual minority men [22], and sexual minority men and women [19] in states adopting same-sex marriage laws or other legal protections for sexual minorities. Because health disparities and poor health may be costly to society [32], policymakers should consider the health benefits of legal protections for sexual minorities when debating new proposals and legislation. Since these data were collected, same-sex marriage has been extended across the United States. While legal same-sex marriage may be associated with improved health for sexual minorities, our study suggests that comprehensive legal protections may be associated with better self-rated health for some LGB people—and the health benefits may not be shared equally. Some sexual minorities may not fully enjoy the health advantages of LGB-affirming policies depending on other sociodemographic factors (e.g., heterosexism, sexism, and racism) or the policy environments found in the places they live (e.g., comprehensive versus limited protections). Our study provides early evidence that sexual minority women still report disparities in self-rated health regardless of their state policy environment—which, for us, was unexpected. Our analysis did not explore the causal mechanisms explaining why disparities in self-rated health persisted for sexual minority women, but the relationship between legal protections and health should be examined from a gendered perspective. Sociologists have found that heterosexual marriage disproportionately improves the health of men compared with that of women (which may be emerging through our findings) [33]. Other sociologists have argued that public policy can reinforce or reshape marital, social, and economic situations for women [34,35]—all of which may affect women’s health differently than men’s health. Future studies should compare the health impacts of LGB-based policies by gender and other sociodemographic dimensions, including race/ethnicity, socioeconomic status, and disability status.

Our research also demonstrates the importance of state-level data for monitoring health disparities among sexual minorities. Having more comprehensive sexual orientation data at the state and municipal levels can inform targeted interventions for improving health outcomes and health behaviors in sexual minorities. Additional data can also help identify groups within the sexual minority population at greatest risk of worse health. For instance, while not examined directly in this study, sexual minority women experienced different trends and disparities in self-rated health compared with sexual minority men. On average, sexual minority women were more likely to report poor/fair self-rated health (20.7%) compared with sexual minority men (17.4%). Relatedly, sometimes disparities in self-rated health between heterosexual individuals and sexual minorities were wider for women than men in the same state (e.g., Iowa, Massachusetts, and Nevada). Future research should use qualitative or mixed-methods study designs to better understand how health is understood or interpreted for sexual minorities and whether definitions of `excellent’ health vary by sexual orientation and within the sexual minority population. Other research should continue to use alternative data (e.g., the National Health Interview Survey) and different settings (e.g., European countries) to confirm or negate these findings.

### Limitations

Our findings should be interpreted with caution, as several limitations were present throughout this study, such as reporting bias, missing data, small sample sizes, and noncausal research designs. First, all responses in the BRFSS—including sociodemographic characteristics and health status—were self-reported, which may suffer from recall and response bias. Relatedly, responses to the sexual orientation question may be threatened by selection bias. The sample of sexual minorities examined in this study only included adults in noninstitutionalized settings (with landline or cell phones) who were comfortable disclosing their sexual orientation to BRFSS surveyors. Homeless adults and adults in nursing homes, incarceration facilities, and institutionalized medical facilities were missing from this study. Also missing were adults not comfortable disclosing their sexual minority status, which may bias our estimates to the extent that vulnerable subpopulations were included among nonrespondents.

Meanwhile, adults in states not using the sexual orientation module in their BRFSS survey were also missing from this analysis. Because our sample only included data from 31 states, our results may not be generalizable to the rest of the country or to the entire sexual minority population. States in the southeastern U.S. were especially underrepresented, which includes numerous states with limited legal protections for LGBT people. Differences in self-rated health may be wider than what we detected here. We are hopeful that additional states will participate in the sexual orientation and gender identity module or that questions on sexual orientation and gender identity will be added to the core BRFSS questionnaire in the near future.

Small sample sizes also restricted our analysis to sexual minority status. We were unable to separate our analysis by specific sexual orientation (i.e., stratified analyses for lesbians, gay men, bisexual men, and bisexual women were less reliable because of small samples). Relatedly, the BRFSS module only asks a single question about sexual orientation identity and does not measure other dimensions of sexual orientation, such as sexual behavior or sexual attraction. Our analysis precludes individuals who are sexually active with or attracted to people of the same sex but do not identify as lesbian, gay, or bisexual. Additionally, our study only focused on health disparities by sexual minority status and did not include comparisons by transgender status or gender identity—both because of the limited sample size of transgender respondents and because gender identity is a separate and distinct concept from sexual orientation [36]. We hope that future research will investigate whether and how self-rated health varies for transgender individuals by state policy environment.

Finally, this analysis is limited by its observational research design. The BRFSS is a cross-sectional health survey and the correlations identified in this analysis cannot definitively establish the causal pathways between sexual minority status, self-rated health, and state policy environments. Omitted and unmeasured variables—such as experiences of sexual-orientation-based discrimination and micro-level exposures to inclusivity or hostility in various settings (e.g., employment, health care, and the household)—may explain the relationship between state policy environments and self-rated health for sexual minorities reported here. Future research should examine the impact of micro-level exposures to discrimination on self-rated health. Other research should use quasi-experimental designs or longitudinal data to test and establish causality in the relationship between legal protections and health outcomes for sexual minorities. Still, more research should build on this study and examine the relationship between legal protections and alternative dimensions of health, including mental health, activity limitations, and health behaviors.

## 5. Conclusions

This study adds new evidence of the association between state policy environments and self-rated health disparities for sexual minorities using a population-based and multistate sample in the United States. While much focus has been placed on the impact of state health policy on health outcomes, this study emphasizes the importance of further understanding the relationships between non-health-related legal protections and the health of populations vulnerable to discrimination. Ultimately, the ability to elucidate the impact of public policies at the federal, state, and local levels will enable the development of better strategies to reduce disparities and improve the overall health of vulnerable populations, including sexual minorities.

## Figures and Tables

**Table 1:** Demographic and socioeconomic characteristics of adult respondents by gender and sexual minority status.

Sociodemographic Characteristics	Men			Women		
	Heterosexual	Sexual Minority	*p* Value	Heterosexual	Sexual Minority	*p* Value
	(*n* = 207,562)	(*n* = 6894)		(*n* = 282,509)	(*n* = 7793)	
Total	96.4	3.6	<0.001	96.2	3.8	<0.001
**Age, years**			<0.001			<0.001
18–24	12.6	21.8		10.7	31.3	
25–34	16.2	21.7		14.7	26.3	
35–44	16.3	12.9		15.6	14.6	
45–54	17.9	18.2		17.7	12.3	
55–64	17.5	14.3		17.7	8.0	
≥65	18.8	10.8		22.8	7.1	
Missing data	0.7	0.2		0.9	0.4	
**Race/Ethnicity**			0.01			<0.001
White	67.1	64.5		67.5	62.6	
Black	10.5	10.8		12.0	13.6	
Hispanic	13.2	14.0		12.5	12.9	
Other/Multiple Races	7.5	9.4		6.7	9.6	
Missing data	1.8	1.3		1.3	1.3	
**Relationship status**			<0.001			<0.001
Married or living with a partner	58.7	32.8		55.2	36.4	
Divorced, separated, or widowed	15.7	10.6		24.6	15.3	
Never married	25.1	55.8		19.8	47.7	
Missing data	0.5	0.8		0.4	0.6	
**Children in the household**			<0.001			0.017
None	64.7	80.8		61.3	58.4	
At least one child	34.8	18.8		38.2	41.3	
Missing data	0.6	0.3		0.4	0.3	
**Educational attainment**			<0.001			0.001
Less than high school	13.3	9.9		11.9	13.6	
High school graduate	30.8	26.3		27.9	26.4	
Some college	29.2	32.3		32.7	35.7	
College graduate	26.4	31.2		27.3	24.1	
Missing data	0.3	0.3		0.2	0.2	
**Employment status**			<0.001			<0.001
Employed	64.5	60.6		50.5	54.9	
Unemployed	5.8	7.7		5.0	9.6	
Not in labor force	29.0	31.1		43.9	34.8	
Missing data	0.7	0.5		0.6	0.8	
**Household income, $**			<0.001			<0.001
0–9999	3.6	5.8		4.9	8.6	
10,000–19,999	9.1	11.8		11.5	14.0	
20,000–34,999	15.6	16.8		17.1	18.8	
35,000–49,999	12.7	13.0		11.6	11.5	
50,000–74,999	14.4	12.4		12.9	10.0	
≥75,000	32.7	28.1		26.7	21.3	
Missing data	12.0	12.0		15.3	15.9	
**Chronic disease diagnoses**			0.06			0.008
None	58.2	60.8		50.6	53.1	
One chronic disease	23.1	21.4		26.7	26.4	
Multiple chronic diseases	15.9	14.7		20.3	17.6	
Missing data	2.8	3.1		2.5	2.9	
**Health insurance status**			0.86			<0.001
Insured	87.4	87.4		90.7	86.8	
Uninsured	12.0	11.7		8.9	12.6	
Missing data	0.6	0.9		0.4	0.6	
**Has usual source of care**			0.85			<0.001
Yes	68.7	69.3		78.9	69.1	
No	30.7	30.2		20.8	30.5	
Missing data	0.6	0.6		0.4	0.4	

Source: 2014–2016 Behavioral Risk Factor Surveillance System.

**Table 2:** Prevalence and odds ratios comparing poor/fair health among men by sexual minority status and state policy environment.

Study Sample	Weighted Prevalence (%)	Unadjusted OR (95% CI)		Adjusted OR (95% CI) ^†^	
**Men in all 31 study states**					
Heterosexual	16.4	1.00	[Reference]	1.00	[Reference]
Sexual Minority	17.4	1.07	(0.95–1.21)	1.10	(0.94–1.28)
**Men in 16 study states with comprehensive protections**					
Heterosexual	15.4	1.00	[Reference]	1.00	[Reference]
Sexual Minority	14.9	0.96	(0.82–1.13)	0.96	(0.79–1.16)
**Men in 15 study states with limited protections**					
Heterosexual	17.4	1.00	[Reference]	1.00	[Reference]
Sexual Minority	20.6	1.23	(1.03–1.47) **	1.30	(1.03–1.64) **

Source: 2014–2016 Behavioral Risk Factor Surveillance System (BRFSS). Notes: OR = odds ratio; CI = confidence interval. ^†^ Adjusted OR estimates are from logistic regression models adjusting for age, race/ethnicity, relationship status, children in the household, educational attainment, household income, employment status, chronic disease status, health insurance status, usual source of care, state of residence, and survey year. *** *p* < 0.001; ** *p* < 0.05.

**Table 3:** Prevalence and odds ratios comparing poor/fair health among women by sexual minority status and state policy environment.

Study Sample	Weighted Prevalence (%)	Unadjusted OR (95% CI)		Adjusted OR (95% CI) ^†^	
**Women in all 31 study states**					
Heterosexual	17.7	1.00	[Reference]	1.00	[Reference]
Sexual Minority	20.7	1.21	(1.09–1.35) **	1.35	(1.17–1.55) ***
**Women in 16 study states with comprehensive protections**					
Heterosexual	16.1	1.00	[Reference]	1.00	[Reference]
Sexual Minority	19.5	1.26	(1.08–1.46) **	1.44	(1.19–1.75) ***
**Women in 15 study states with limited protections**					
Heterosexual	19.2	1.00	[Reference]	1.00	[Reference]
Sexual Minority	22.0	1.19	(1.01–1.40) **	1.28	(1.04–1.57) **

Source: 2014–2016 Behavioral Risk Factor Surveillance System (BRFSS). Notes: OR = odds ratio; CI = confidence interval. ^†^ Adjusted OR estimates are from logistic regression models adjusting for age, race/ethnicity, relationship status, children in the household, educational attainment, household income, employment status, chronic disease status, health insurance status, usual source of care, state of residence, and survey year. *** *p* < 0.001; ** *p* < 0.05.
